# The interplay between Epstein-Barr virus DNA and gut microbiota in the development of arthritis in a mouse model

**DOI:** 10.1128/spectrum.02042-23

**Published:** 2023-08-24

**Authors:** Sukayna Fadlallah, Elio R. Bitar, Hadi Hussein, Mary-Ann Jallad, Ghassan M. Matar, Elias A. Rahal

**Affiliations:** 1 Department of Experimental Pathology, Immunology, and Microbiology, American University of Beirut, Beirut, Lebanon; 2 Center for Infectious Diseases Research, American University of Beirut, Beirut, Lebanon; University of Florida College of Dentistry, Gainesville, Florida, USA

**Keywords:** rheumatoid arthritis, C57BL/6J mice, gut microbiota, Epstein-Barr virus DNA, proinflammatory responses

## Abstract

Epstein-Barr virus (EBV) DNA may influence the development of autoimmune diseases by increasing the production of proinflammatory cytokines. Such cytokines have been associated with inducing the dysbiosis of colonic microbiota, which, in turn, is a risk factor for autoimmune diseases such as rheumatoid arthritis (RA). Therefore, we investigated the role that EBV DNA may play in modulating the intestinal microbiota and consequent exacerbation of arthritis in a mouse model. Mice were treated with collagen (arthritis-inducing agent), EBV DNA and collagen, EBV DNA, or water. Fecal samples were collected from arthritic and control mice, and 16S rRNA sequencing was performed to determine the effect of EBV DNA on the composition of colonic microbiota. EBV DNA causes a change in the alpha diversity of the microbiota resulting in an increased Chao1 microbial richness and decreased Shannon diversity index in the RA mouse model. In addition, the abundance of particular genera/genus clusters was significantly altered among the various groups, with the EBV DNA-exacerbated arthritic group having the highest number of altered genera/genus cluster abundances. This group also had the highest number of cells co-expressing IL-17A, FOXP3, and IFNγ in the colons. Antimicrobial-cleared mice transplanted with fecal samples from EBV DNA-exacerbated arthritic mice showed a higher incidence and enhanced severity of RA compared to those transplanted with fecal samples from water or collagen-treated mice.

**IMPORTANCE:**

Epstein-Barr virus (EBV) DNA alters the composition and diversity of the gut microbiota in a rheumatoid arthritis (RA) mouse model. These induced changes are associated with enhanced severity of symptoms. This better understanding of the various factors involved in the development of RA will possibly help in creating individualized treatments for RA patients including target mediators triggered by viral DNA. Given that a large swathe of the population harbors EBV, a significant proportion of subjects with arthritis may benefit from possible approaches that target EBV or mediators triggered by this virus.

## INTRODUCTION

Rheumatoid arthritis (RA) is a chronic inflammatory disorder that affects 1% of the world’s population (1). The pathogenesis of RA is not fully understood; however, several risk factors including genetic predisposition and environmental challenges such as alterations in gut microbiota and infectious agents can act as a trigger (2, 3). The gut contains the largest density of microbial residents and plays a role in vitamin synthesis, nutrient absorption, and protection from opportunistic infections (4). Furthermore, the composition of the colonic microbiota can either maintain a hemostatic state or induce inflammation by shaping the immune system. In a healthy gut, the microbiota plays a role in the regulation of certain immune cell populations such as Th17 and Treg cells, thus affecting immune responses, development of autoimmune diseases, and immune tolerance (5). Under certain pathological conditions or environmental triggers, the balance in the microbiota composition is altered leading to dysbiosis. This results in dysregulated immune responses and inflammation which can lead to tissue damage and disease development (6).

Several studies have been carried out to identify the alterations of the gut microbiota in RA. One study showed that *bifidobacteria, Bacteroides–Porphyromonas–Prevotella* group, *Bacteroides fragilis* subgroup, and *Eubacterium rectale–Clostridium coccoides* were less abundant in RA patients (7). Another study found that Lactobacillus spp. was more abundant in RA patients during the early stages when compared to healthy controls (8). Studies in mice illustrated that certain members of the gut microbiota such as segmented filamentous bacteria (SFB) or Lactobacillus can lead to a localized immune response, such as the secretion of interleukin (IL)-6, IL-23p19, tumor growth factor (TGF)-β, and induction of naïve CD4+ T cells to differentiate into Th17. Subsequently, autoreactive T cells promote B-cell differentiation into plasma cells, thus producing autoantibodies. Effector cells such as fibroblasts, osteoclasts, proteases, and macrophages are activated as the self-antibodies and autoreactive T cells become localized to the synovial tissue. This can eventually lead to rheumatoid arthritis (9).

Infectious agents such as Epstein-Barr Virus (EBV) have also been linked to RA development and progression. EBV belongs to the *Herpesviridae* family and is considered to be one of the most prevalent viruses that affect humans (10, 11). This virus usually establishes latency during which it can reactivate resulting in the production of viral DNA. This DNA is capable of immunomodulation (12) possibly altering susceptibility to other diseases. Previous research by our group showed that EBV DNA induces proinflammatory responses in mice and that the EBV DNA load in patients with RA corresponds to a higher serum level of IL-17A, a proinflammatory cytokine (13, 14). In addition, a recent study by our group showed that EBV DNA enhances the incidence and severity of RA in a collagen-induced arthritis (CIA) mouse model (15). Hence, in this study, we intended to determine the effect of EBV DNA on the composition and diversity of intestinal microbiota and identify whether this plays a role in arthritis development in this mouse model.

## MATERIALS AND METHODS

### Mice

The murine model for arthritis employed was collagen-induced arthritis in C57BL/6J mice (16). Female mice of 12 wk old were utilized. The mice were obtained from the animal care facility at the American University of Beirut and treated according to the Institutional Animal Care and Use Committee guidelines. They were co-housed in nonindividually ventilated cages (non-IVC) in the same room and had access to unlimited water and food. Induction of arthritis was carried out as previously described in a recent study (15). The study included an arthritis control group that received the inducing agent and the booster. One group was administered EBV DNA (Advanced Biotechnologies, Columbia, MD) 6 days prior to the primary challenge with collagen. This was selected for administration of EBV DNA based on our previous observations that the pro-autoimmune cytokine IL-17A peaks in mouse sera 6 days after administration of the DNA (13). Hence, in the current study, the DNA was administered, so peak levels of IL-17A would either coincide with collagen administration. Mice that received distilled water alone or 144 × 10^3^ copies of EBV DNA in 100 µL of distilled water intraperitoneally were also examined. Injections containing the viral DNA harbored 144 × 10^3^ copies of EBV DNA in 100 µL of distilled water and were given intraperitoneally. Mice were then monitored for 70 days for the development of arthritis by assessing the ankle joint macroscopically for redness and swelling.

### DNA extraction

At the end of the monitoring period, fresh stool samples were collected from the various groups of mice and stored immediately at −80°C for microbiota analysis and future fecal transplantation experiments. Total genomic DNA was extracted from the stool samples of 8–12 mice per group using the phenol-salt precipitation method. Initially, the stool pellets were thoroughly homogenized using a tissue homogenizer (Dremel, Wisconsin, USA) until no solid particles were visible. Phenol was then added at a volume of 250 µL to the suspension and vortexed to ensure maximum DNA concentration. The samples were centrifuged at a high speed for 15 min at 4°C. The upper layer was collected and 3M sodium acetate (pH: 5.2), glycogen, and 100% ethanol were added. The mixture was then stored at −80°C overnight. The following day, the samples were centrifuged at a high speed for 15 min at 4°C and the pellet was washed three times using 70% ethanol to remove impurities. Finally, the samples were resuspended using Tris-EDTA (TE, pH 8.0). The concentration of the DNA was measured using the DeNovix DS-11 (Wilmington, USA).

### Sequencing

After carrying out quantity and quality checks, 16S rRNA sequencing was carried out. The sequencing library was prepared primers which results in an amplicon whose size is around 400–450 bp. Library construction involved random fragmentation of the DNA sample combined with 5′ and 3′ adapter ligation. The adapter-ligated fragments were then PCR amplified and gel purified. The DNA samples were amplified using the universal primers that target the 16S region (v3-v4): Bakt_341F: CCTACGGGNGGCWGCAG and Bakt_805R: GACTACHVGGGTATCTAATCC. For cluster generation, the library was loaded into a flow cell where the generated fragments were acquired on a lawn of surface-bound oligos complementary to the library adapters. This resulted in the generation of distinct, clonal clusters through bridge amplification. When cluster generation was complete, the templates were sequenced using the Herculase II Fusion DNA Polymerase Nextera XT Index Kit V2 (Aligent, CA, USA) and the Next Generation Sequencing (NGS) Illumina MiSeq platform.

### Microbiota analysis

Raw data were analyzed using the useGalaxy.org platform. Initially, raw data were uploaded as FASTQ files onto the usegalaxy platform. This was followed by the quality control step. After assessing and improving the quality of the data, sequence alignment was carried out. This was followed by data cleaning, preclustering, and removal of the chimera. After cleaning the data, taxonomic classification was carried out and nonbacterial sequences were removed. Finally, Operational Taxonomic Unit clustering was carried out. The percent abundance of the phyla and genera identified in each group was determined and the relative abundance of the microbiota was determined in each group. This was done by identifying the abundance of the major phyla and genera/genus clusters, characterized as having a mean abundance of >1% of the total bacteria.

Diversity index calculations were then carried out to determine the alpha and beta diversity. Alpha diversity was determined in terms of the Shannon index and the Chao1 index which represent the abundance/evenness and the richness of the data, respectively. The Shannon index was calculated using the formula: Shannon Index (*H*) = − Σ p ln p [where p is the proportion (*n*/*N*) of individuals of one particular species/genus (*n*) divided by the total number of individuals (*N*)]. The Chao1 index was calculated using the formula: Chao1 = *S*_obs_ + *f*1(*f*1−1)/2(*f*2+1) (where *S*_obs_ is the number of species/genera observed, *f*1 is the number of singletons and *f*2 is the number of doubletons) (17). The beta diversity was calculated in terms of principle coordinate analysis (PCOA), which determines the dissimilarity between the data sets. The PCOA was calculated using XLSTAT 2014 and was based on the Bray and Curtis dissimilarity distance (18).

### Immunofluorescence staining for mouse colon tissues

The number of cells that were double positive for IL-17A^+^/ interferon gamma ((IFNγ^+^) or IL-17A^+^/FOXP3^+^ and triple positive for IL-17A^+^/ IFNγ^+^ / FOXP3^+^ in colons from the various groups of mice was determined by immunofluorescence (IF) performed on histological sections of the colons. The immunofluorescence was carried out as previously described (15); however, the colon sections did not undergo the decalcification step. The antibodies used were fluorochrome-linked Brilliant Violet 605 antimouse IL-17A (1:1500), Pacific Blue 405 antimouse IFNγ (1:1,500), and Alexa Fluor 488 antimouse FOXP3 (1:1500) (Biolegend, California, USA). The number of double-positive cells per area was determined using ImageJ (NIH, Wisconsin, USA) and is expressed as count per inch^2^.

### Fecal transplantation from EBV DNA-treated arthritic mice

To determine whether the gut microbiota contributes directly to arthritis progression by fecal transplantation from EBV arthritis-affected mice to antimicrobial-treated mice, 66 female, 3-wk-old C57BL/6J mice were used. The pups were obtained as soon as they could be weaned from their mothers which occurs at week 3 of age. The mice were then divided into three groups each containing 22 mice. The mice received an antimicrobial cocktail that consisted of amoxicillin (0.5 mg/mL), vancomycin (2.5 mg/mL), metronidazole (0.5 mg/mL), amphotericin B (0.025 mg/mL), streptomycin (0.025 mg/mL), penicillin (25 U/mL), and ciprofloxacin (0.0625 mg/mL) (19). The antimicrobial cocktail was supplied in drinking water on alternate days for 5 wk. Confirmation of the depletion of microbiota from the gut of the mice was done by culturing the feces obtained at the end of the 5-wk period on different agar media plates. These plates included the following: trypticase soy agar, blood agar, MacConkey agar, International Streptomyces Project Medium 3 agar (oatmeal agar), and Luria-Bertani agar. The absence of bacterial colonies on these plates validated the loss of microbiota from these mice. Subsequently, the mice received fecal samples previously obtained from (1) distilled water group, (2) collagen-treated arthritic mice, and (3) EBV DNA 6 days prior to collagen-treated arthritic mice. Fecal suspensions were prepared by homogenizing one pellet (~20 mg) in sterile phosphate-buffered saline per mouse using a tissue homogenizer. The homogenate (200 µL) was given to each mouse by oral gavage. Fecal microbiota transplantation was done once per week for 4 wk. Collagen-induced arthritis was then carried out as previously described to induce the disease.

### Evaluation of the incidence and severity of arthritis

The mice were monitored for 70 days for the development of arthritis by assessing the ankle joint macroscopically for redness and swelling. At the end of the monitoring period, the severity of arthritis was determined in terms of hind paw thickness, grip strength, clinical scoring, and histological scoring of ankles and footpads as previously described (15).

### Statistical tests

Statistical analysis was performed using Graphpad Prism v6. The comparisons in the histological and clinical scores were done using the Mann–Whitney *U* test. Mean comparisons and alpha diversity indices were analyzed using the two-tailed unpaired Student’s *t*-test. *P* values less than 0.05 were considered statistically significant.

## RESULTS

### EBV DNA alters the composition and diversity of intestinal microbiota in the RA mouse model

**Fig 1:**
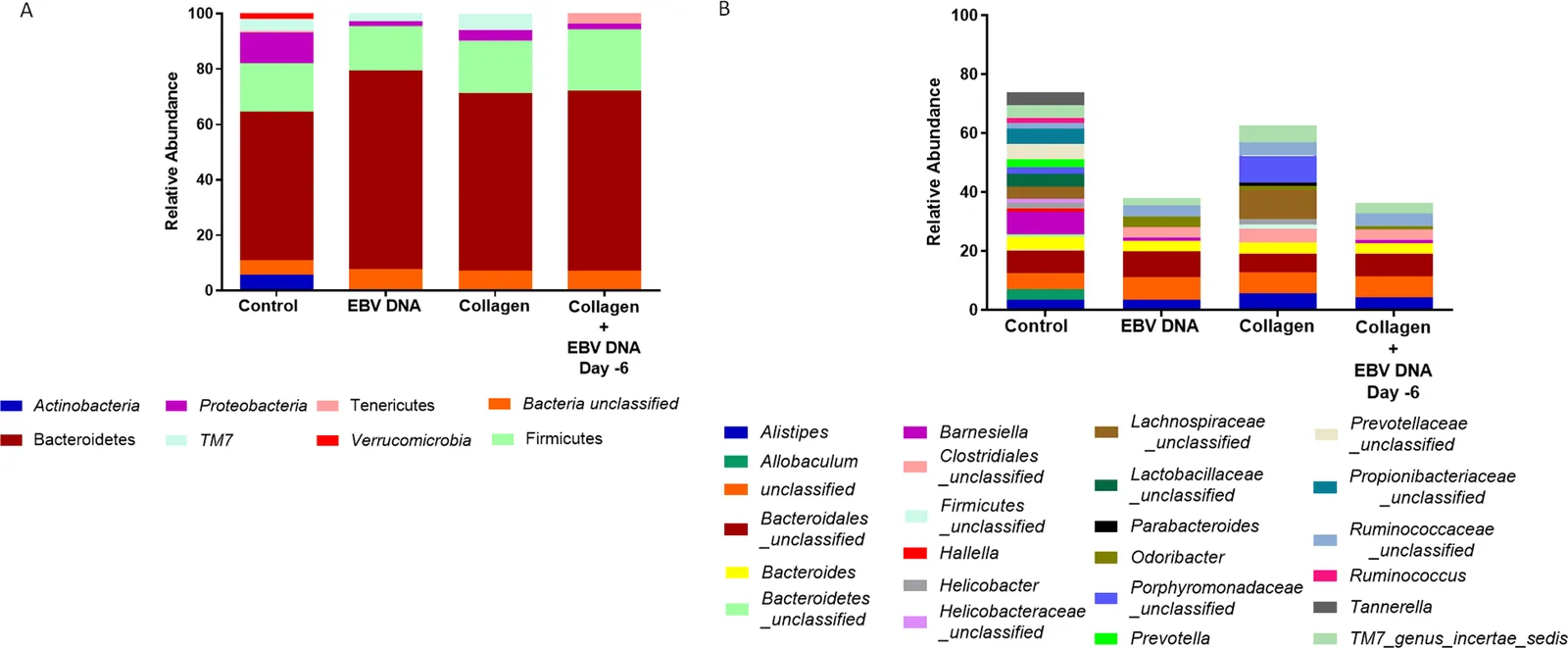
Relative abundance of colon microbiota phyla and genera/genus clusters detected in arthritic and control mice in the EBV DNA-treated collagen-induced arthritis mouse model. (**A**) Relative abundance of the major bacterial phyla and (B) relative abundance of the major bacterial genera/genus, characterized as having a mean abundance of >1% of the total bacterial content in groups that received distilled water, EBV DNA only, collagen only, or EBV DNA 6 days prior to collagen determined using 16S rRNA sequencing. Data are mean abundance expressed as the percentage of the total bacterial count.

The relative abundance of the major phylum and genera/genus clusters, characterized as having a mean abundance of >1% of the total bacteria, was identified in the arthritic and control mice (Fig. 1A). Seven different phyla were present in the different groups with varying abundance. All groups were predominated by the *Bacteriodetes* phylum with the highest percentage being present in the group that received EBV DNA. *Actinobacteria*, *Probacteria*, and *Verrucomicrobia* phyla were highest in the group that received distilled water and drastically decreased in the remaining groups. *Tenericutes* and *Firmicutes* phyla were highest in the group that received collagen and EBV DNA. Finally, the group that received collagen only had the highest percentage of *Tenericutes* phyla.

The microbiota of control samples that received distilled water was predominated by the genera/genus clusters (most abundant to least abundant) *Barnesiella, Bacteriodes, Tannerella, TM7 genus incertae sedis, Allobaculum, Alistipes, Prevotella, Ruminococcus, Helicobacter*, and *Hallela*. On the other hand, the microbiota of the group that was treated with collagen only was distinct and comprised mainly of *Alistipes, TM7 genus incertae sedis, Bacteriodes, Helicobacter, Odoribacter*, and *Parbacteriodes*. The microbiota of groups that were treated with either EBV DNA only or EBV DNA 6 days prior to collagen contained the same core genera/genus clusters and contained *Bacteriodes, Alistipes, TM7 genus incertae sedis, Barnesiella*, and *Odoribacter* (Fig. 1B)

**Fig 2:**
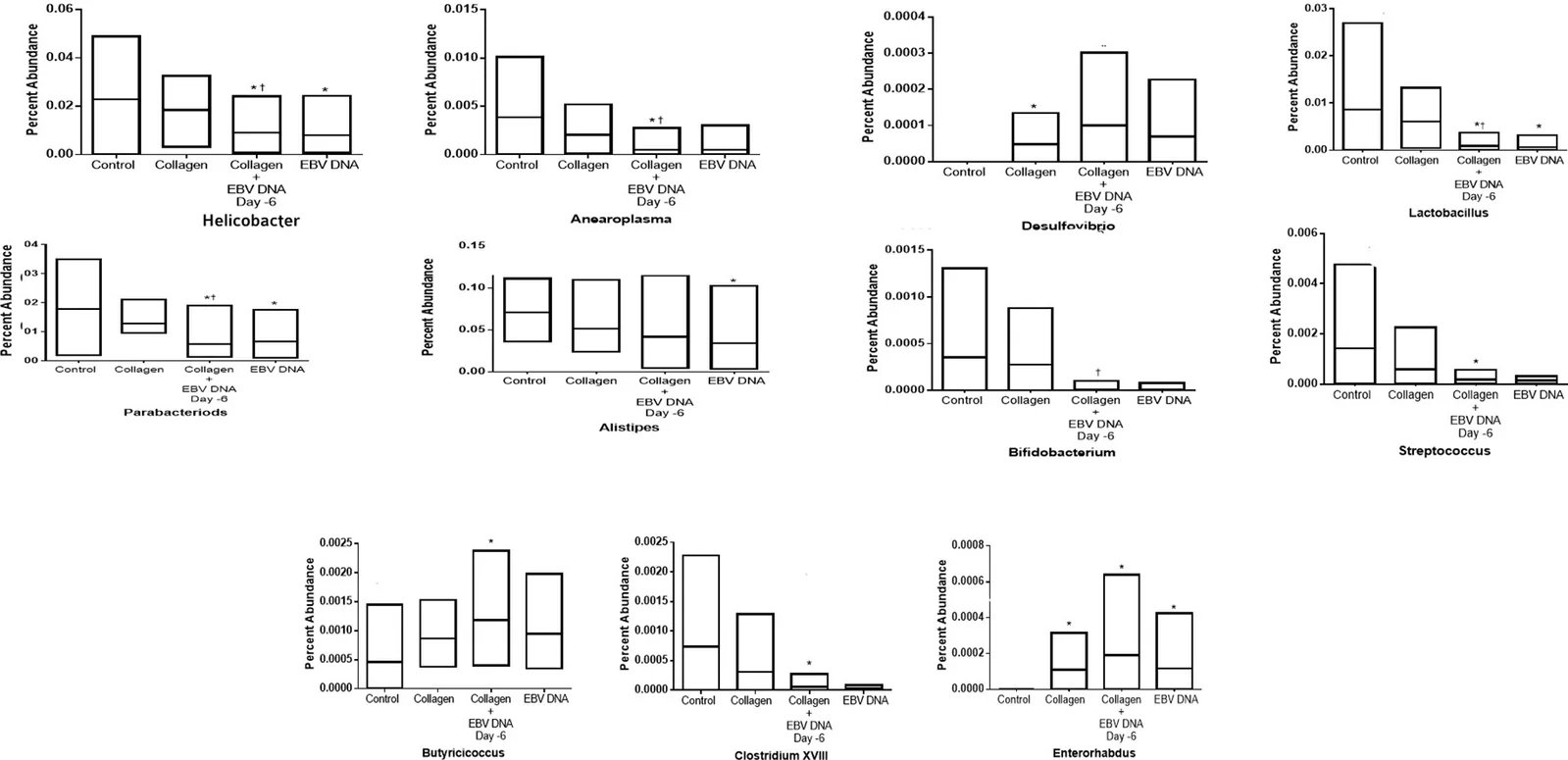
The percent abundance of microbiota genera/genus clusters whose abundance was altered in arthritic and control mice in the EBV DNA-treated collagen-induced arthritis mouse model. The percent abundance of genera/genus clusters that were significantly altered in mouse groups that received distilled water, EBV DNA only, collagen only, or EBV DNA 6 days prior to collagen determined using 16S rRNA sequencing. * indicates *p*˂ 0.05 compared to mice treated with distilled water only. †indicates *p*˂ 0.05 compared to mice treated with collagen only.

We identified that the percent abundance of 33 genera/genus clusters was significantly altered among the groups that received collagen alone, EBV DNA 6 days prior to collagen, EBV DNA, or distilled water. The genera/genus clusters that showed a trend of increased or decreased percent abundance in both arthritic groups were identified. An increase or decrease in the same direction of the group that received EBV DNA 6 days prior to collagen might provide insight into the genera/genus clusters that could play a role in arthritis and explain the differences in the severity of the disease among the arthritic groups. Among the 33 genera that were significantly altered among the various groups in comparison to the control, 26 showed a trend in the change in the percent abundance (Figure and). Among these genera/genus clusters, six showed an increase in the percent abundance in arthritic groups in comparison to the distilled water group namely *Desulfovibrio, Enterorhabdus, Paraprevotella, Pseudomonas, Veillonella, and Butyricicoccus*. The remaining 20 genera/genus clusters showed a decrease in the percentage abundance in mice that had arthritis in comparison to the control mice. These included *Acinetobacter, Anearoplasma, Clostridium cluster XVIII, Enhydrobacter, Helicobacter, Lactobacillus, Methylobacterium, Micrococcus, Mucispirillum, Nitrospira, Orbus, Parabacteriods, Paracoccus, Perlucidibaca, Propionibacterium, Rhodococcus, Rothia, Streptococcus, Alistipes*, and *Bifidobacteria*. The percentage abundance of the number of selected genera significantly altered between the various groups and is shown in Fig. 2.

**Fig 3:**
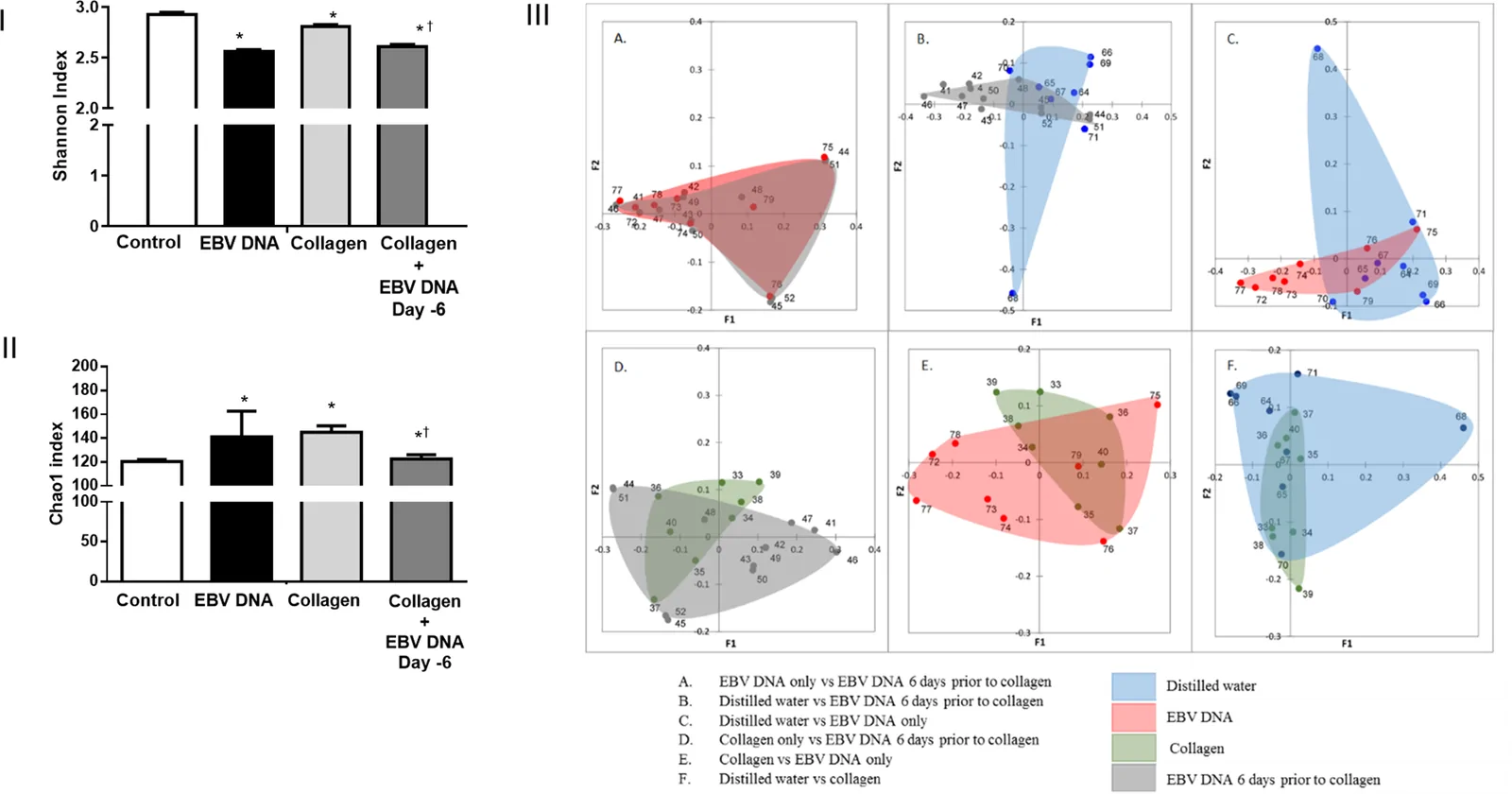
Diversity indices of mouse colon microbiota in the EBV DNA-treated collagen-induced arthritis mouse model. (**I**) Shannon index and (II) Chao1 richness estimator index as a measure of alpha diversity in mouse groups that received distilled water, EBV DNA only, collagen only, or EBV DNA 6 days prior to collagen (III) PCOA based on the Bray and Curtis dissimilarity distance as a measure of beta diversity, comparisons were made between groups that were treated with distilled water, EBV DNA only, collagen only, or EBV DNA 6 days prior to collagen. * indicates *p*˂ 0.05 compared to mice treated with distilled water only. † indicates *p*˂ 0.05 compared to mice treated with collagen only.

We measured the Shannon and Chao1 indices of the microbiota among the various groups to determine the alpha diversity. The Shannon index is an estimator of diversity in terms of abundance, evenness, and richness (20). The Choa1 index, on the other hand, is an estimator of the richness in a community. It is based on the notion that rare species provide the most information about the number of missing species (21). Results showed that the Shannon index significantly decreased in all groups in comparison to the distilled water group (p ≤ 0.0001 in all groups) (Fig. 3I). The greatest decrease occurred in the group that received EBV DNA only. The Chao1 index increased significantly in all groups when compared to the distilled water group (p value ≤ 0.0001 in all groups) (Fig. 3II). The highest increase in the richness occurred in the group that received collagen only. In addition, both the Shannon and Chao1 indices significantly decreased in the group that was treated with EBV DNA 6 days prior to collagen in comparison to the group that was treated with collagen only (p ≤ 0.0001 for both indices).

On the other hand, beta diversity was calculated using the PCOA. Our results showed that the beta diversity was not dissimilar among the various groups (Fig. 3III). The samples in the group that received EBV DNA almost overlapped with the samples in the group that received EBV DNA 6 days prior to collagen.

### EBV DNA increases double-positive IL-17A/ IFNγ, double-positive IL-17A/ FOXP3 cells, and triple-positive IL-17A/ FOXP3/IFNγ cells in colon tissues of arthritic mice

**Fig 4:**
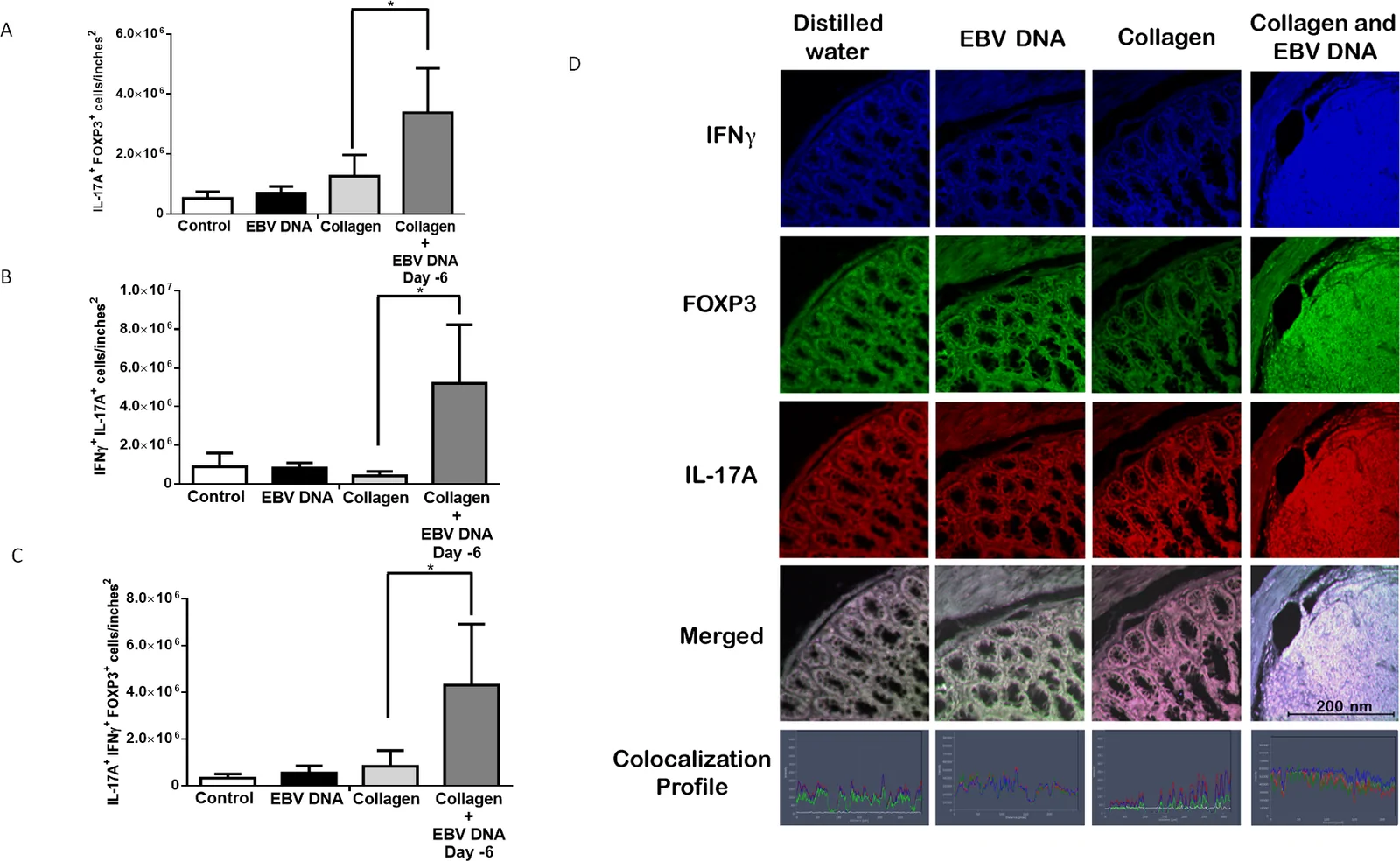
Immune cell populations in colons from the EBV DNA-treated collagen-induced arthritis mouse model. (**A**) IFNγ ^+^IL-17A^+^, (**B**) IL-17A^+^ FOXP3^+^, and (**C**) IL-17^+^ FOXP3^+^ IFNγ ^+^cell counts were determined by immunofluorescent staining of colon sections from arthritic animals in C57BL/6J mouse groups treated with distilled water, EBV DNA only, collagen only, or EBV DNA 6 days before collagen; * indicates *p*˂ 0.05 compared to mice treated with collagen alone. (**D**) Immunofluorescent staining for IL-17A, FOXP3, and IFNγ in colon sections from C57BL/6J mouse groups treated with distilled water, EBV DNA only, collagen only, or EBV DNA 6 days before collagen.

Under pathological conditions, the intestinal microbiota is altered leading to dysbiosis. The result is dysregulated immune responses and inflammation in the colon, which can eventually lead to tissue damage and disease development. Hence, to determine part of the dysregulated immune responses, the number of double-positive IL-17A/ IFNγ cells, double-positive IL-17A/ FOXP3 cells, and triple-positive IL-17A/ FOXP3/IFNγ cells was identified in the colons of the various groups of mice with immunofluorescence staining. The number of IL-17A+/IFNγ+ cells was the highest in the colons of mice that received EBV DNA 6 days prior to collagen (*p* = 0.0147 vs control, 0.0457 vs collagen, and 0.0122 vs EBV DNA only) (Fig. 4A). The lowest number of L-17A^+^/IFNγ^+^ cells was observed in the colons of mice that received collagen only; however, this decrease was not significant in comparison to the control (*p = 0.19 vs control and 0.832 vs EBV DNA) (Fig. 4B). Similarly, the number of IL-17A^+^ FOXP3^+^ cells in the colons was highest in mice that received EBV DNA 6 days prior to collagen (*p* = 0.0178 vs control, 0.00205 vs collagen, and 0.0039 vs EBV DNA only). The number of IL-17A^+^ FOXP3^+^ in the colons of mice that were treated with collagen only was increased in comparison to the control (*p* = 0.054) (Fig. 4B). When the number of triple-positive IL-17A/ FOXP3/IFNγ cells was determined in the various groups in the colon sections, it was observed that the highest number of these cells was found in mice treated with EBV DNA 6 days prior to collagen (*p* = 0.01 vs control, 0. 0.0207 vs collagen, and 0.0128 vs EBV DNA only) (Fig. 4C). The number of triple-positive IL-17A/ FOXP3/IFNγ cells in colon sections of mice treated with collagen only was higher than that of mice treated with EBV DNA only or distilled water; however, this increase was not significant (*p* = 0.173 vs control and 0.411 vs EBV DNA). Figure 4D shows immunofluorescent staining for IL-17A, IFNγ, and FOXP3 in arthritic mice and control mice

### Fecal microbiota transplantation from EBV arthritis-affected mice to antimicrobial-treated mice shows that microbiota contributes directly to arthritis progression

To determine whether the microbiota composition contributes directly to arthritis progression in an RA mouse model, fecal transplantation from EBV arthritis-affected mice to antimicrobial-treated mice was carried out.

**Fig 5:**
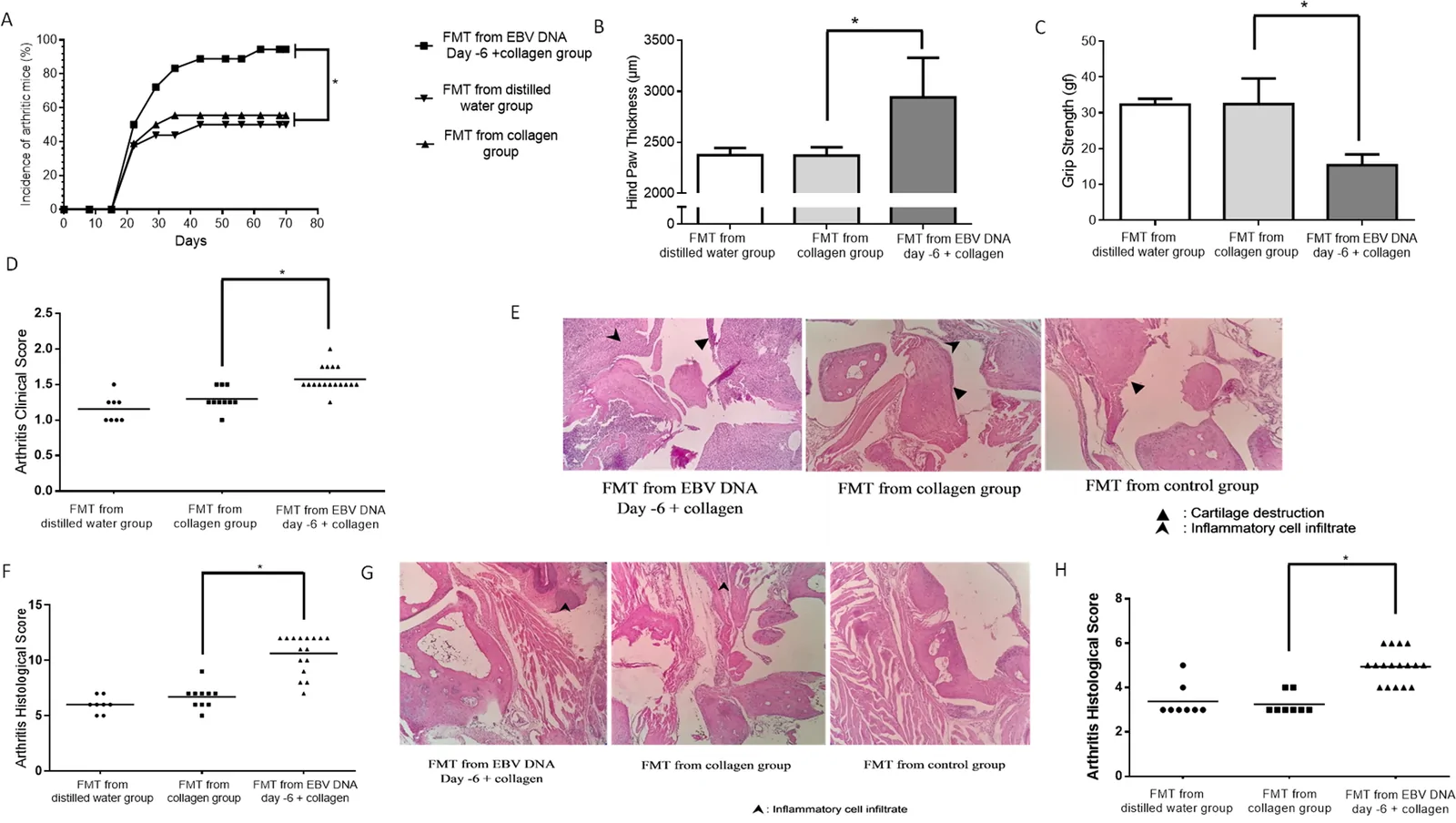
Fecal microbial transplantation (FMT) in the collagen-induced arthritis mouse model. (**A**) Incidence of arthritis in mice that received FMT from EBV DNA-treated arthritic mice and subsequently induced with type II chicken collagen. Incidence (%) of arthritis in various groups of C57BL/6J mice that received feces from mice that were injected with type II chicken collagen (*n* = 18), mice that were treated with EBV DNA 6 days before collagen (*n* = 18), or from mice treated with distilled water (*n* = 16). After FMT, arthritis was induced by type II chicken collagen administration; (**B**) average hind paw thickness, (**C**) average grip strength, (**D**) clinical scores, (**E**) representative histological sections of ankle joints, (**F**) histological scores of the ankle, (**G**) representative histological sections of footpads, and (**H**) histological scores of the footpads, in various groups of C57BL/6J mice that received FMT from mice that were injected with collagen alone, mice that were treated with EBV DNA 6 days before collagen, or mice treated with distilled water. After FMT, arthritis was induced by type II chicken collagen administration; * indicates *p*˂ 0.05 compared to the group that received feces from mice treated with collagen only.

Three groups of mice were transplanted with feces obtained previously from mice that received distilled water, collagen only, or EBV DNA 6 days prior to collagen. These mice were then induced with type II chicken collagen to develop arthritis. The incidence of arthritis in the mice that were inoculated with feces from the group that was previously treated with collagen only was 55.5%. Similarly, the group of mice that were given feces from mice that received distilled water had a 50% incidence of the disease. However, when mice were given feces from the group that was previously treated with EBV DNA 6 days prior to collagen, the incidence of arthritis significantly increased (94.4%, *p* = 0.0178 compared to the group that received feces obtained previously from mice that were given collagen, *p* = 0.0056 compared to the group that received feces obtained previously from mice that were injected with distilled water). All of these mice started developing arthritis at day 22 postinduction with collagen. Figure 5A shows the incidence of arthritis in the groups of mice that were subjected to fecal transplantation and arthritis induction.

The average hind paw thickness in mice that received feces from collagen-treated mice and mice that were inoculated with feces from distilled water-injected mice was similar. However, when mice were given feces from mice that were treated with EBV DNA 6 days prior to collagen, the hind paw thickness significantly increased, an indication of increased severity of arthritis (*p* ˂0.0001 compared to mice that received feces from collagen-treated mice). Figure 5B shows the average hind paw thickness in the various groups of mice. The grip strength of the affected paw in arthritic mice was then measured to assess the function of the joint and determine the severity of arthritis. The average grip strength in the group that received feces from distilled water-injected mice and the group that was given feces from collagen-injected mice was similar. The average grip strength significantly decreased in mice that received feces from EBV DNA and collagen-treated mice (*p* ˂0.0001 compared to mice that received feces from collagen-treated mice) (Fig. 5C).

The clinical score of the mice that developed arthritis after collagen induction and fecal transplantation from the various groups was determined. The arthritic clinical scores of mice that received feces from EBV DNA in addition to collagen-injected mice were significantly higher than the clinical scores of mice that received feces from mice that were treated with collagen or distilled water (*p* = 0.0006 compared to mice that received feces from collagen-treated mice and *p* ˂0.0001 compared to mice that received feces from distilled water-injected mice) (Fig. 5D). A high proportion (87.5% and 70%) of the clinical scores were below a score of 1.5 in the groups that received feces from mice that were treated with collagen and mice that were injected with water respectively. However, 94% of the clinical scores clustered at a score of 1.5 and above in mice that received feces from mice that were given EBV DNA 6 days prior to collagen.

The histological scores of the ankle joints and footpads were determined in arthritic mice that were subject to fecal transplantation from the various group of mice described above to assess tissue damage and determine the severity of inflammation. The histological scores of the ankle joints of arthritic mice in the group that was transplanted with feces from mice that received EBV DNA 6 days prior to collagen were significantly higher than the scores of mice that received feces from mice that were distilled water treated or collagen injected (*p* ˂0.0001 compared to mice that received feces from distilled water-injected mice and mice that received feces from collagen-treated mice) (Fig. 5E). The histological scores of the ankle joints in the arthritic mice were all below a score of 8 in mice that received feces from distilled water-injected mice. Similarly, a large proportion (90%) of the scores were clustered below a score of 8 in arthritic mice that received feces from collagen-treated mice. On the other hand, 94% of the histological scores were a score of 8 or above in the arthritic mice that were inoculated with feces from mice that received EBV DNA 6 days prior to collagen.

The histological scores of the footpads in arthritic mice were significantly higher in the group that was given feces from mice that received EBV DNA 6 days prior to collagen than the scores of mice that received feces from mice that were distilled water treated or collagen injected (*p* ˂0.0001 compared to mice that received feces from collagen-injected mice and *p* = 0.0003 compared to mice that received feces from distilled water-treated mice) (Fig. 5G). A quarter of the histological scores of the footpads in the arthritic mice were a score of 4 and above in mice that received feces from distilled water-injected mice and mice that received feces from collagen-treated mice. On the other hand, all of the histological scores were above a score of 4 in the arthritic mice that were transplanted with feces from mice that received EBV DNA 6 days prior to collagen. These results show that the level of inflammation and damage was highest in arthritic mice that received feces from mice treated with EBV DNA 6 days prior to collagen. Figure 5E and G shows representative histological sections from ankle joints and footpads of arthritic mice that received feces from the various groups.

## DISCUSSION

In this study, we assessed the effect of EBV DNA on the composition, diversity, and abundance of the microbiota in an RA mouse model. The group that had the largest number of genera whose percent abundance was significantly altered compared to the control was the group that received EBV DNA 6 days prior to collagen. The group that received collagen only had a fewer number of genera whose percent abundance was significantly changed in comparison to the control. This was also supported by the Shannon index which showed that the diversity of the microbiota was significantly decreased in arthritic mice in the groups that were treated with collagen alone or treated with EBV DNA 6 days prior to collagen. This is in accordance with other studies that showed the diversity in terms of the Shannon index was decreased in arthritic mice (22, 23). These results are also in accordance with other studies that characterized the gut microbiota in human autoimmune diseases such as RA, inflammatory bowel disease (IBD), and psoriasis (24 – 26). Our results also showed that the decrease in the diversity of the microbiota was more pronounced in arthritic mice that were treated with EBV DNA 6 days prior to collagen, the group of mice that showed the highest severity and incidence of arthritis. A study by Chen et al. (27) showed that the alpha diversity of microbiota in terms of the Shannon index negatively correlates with rheumatoid factor levels (indicating disease severity) in RA patients (27). On the other hand, the Chao1 index, which represents the richness of the microbiota in terms of rare species, was significantly increased in arthritic mice that received collagen when compared to the control group. This may be due to the decreased abundance of several genera/genus clusters in the EBV DNA-treated mice, which would allow over-representation of rare species. Concerning the beta diversity, our results showed that samples from the different groups were not dissimilar and diverse. This is not surprising as differences in the abundance of a small number of genera will not likely cause divergence in the composition of the microbiota.

The percent abundance of *Butyricicoccus*, *Enterorhabdus*, and *Desulvovibrio* was increased in mice that had arthritis in comparison to the control and this increase was highest in the group that received EBV DNA 6 days prior to collagen. A study employing the DBA/1 CIA mouse model showed that the abundance of the *Enterorhabdus* genus was low in mice prior to arthritis development but dramatically increased as the disease progressed. In the same study, the abundance of the *Desulvovibrio* genus was also increased in arthritic DBA/1 mice in comparison to control mice (28). In our study, we showed that the abundance of the genus *Butyricicoccus* was increased in arthritic mice in comparison to the control. *Butyricicoccus*, belonging to the family of *Clostridiaceae*, is a producer of butyrate, a short-chain fatty acid (SCFA). SCFAs help promote epithelial function and induce anti-inflammatory immune responses in the intestines (29). The abundance of *Butyricicoccus* in our study may have increased in arthritic mice due to the changes in the flora composition; however, this increase was not sufficient to compensate for the other proinflammatory factors.

The percent abundance of *Helicobacter*, *Parabacteriods*, *Alistipes*, *Anearoplasma*, *Lactobacillus*, *Bifidobacterium, Clostridium XVIII*, and *Streptococcus* was significantly reduced in arthritic mice in comparison to the distilled water-treated mice. The greatest decrease in the abundance of these genera was in the group of mice injected with EBV DNA 6 days prior to collagen. The role of *Anearoplasma, Clostridium XVIII, Parabacteriods*, and *Bifidobacteria* as immunomodulatory agents in the gut microbiota has been well documented. A recent study by Beller et al. (30) demonstrated that gut microbiota belonging to the genus *Anearoplasma* increases the levels of mucosal IgA, strengthens the intestinal barrier, and promotes anti-inflammatory immune responses by inducing TGF-β production (30). Similarly, Atarashi et al. (31) showed that Clostrdia species including the *Clostridium XVIII* cluster induce the production of IL-10 and promote Treg proliferation (31). In addition, the abundance of *Clostridium XVIII* has been identified to be low in autoimmune diseases such as RA (32). Another genus that has been associated with anti-inflammatory immune responses is *Parabacteroids*. A study by Kverka et al. (33) showed that *Parabacteroids distasonis* drives T-cell differentiation toward an anti-inflammatory immune response (33). Species of *Bifidobacterium* genus are normal residents of a healthy human intestine and modifications in their composition or a decrease in the abundance of their populations is one of the most frequent characteristics present in inflammatory and autoimmune diseases (34). These are in line with our results, which show that the percent abundance of these genera was significantly reduced in arthritic mice in comparison to control mice. The proportions of the *Alistipes* and *Streptococcus* genera were reduced in our study in arthritic mice when compared to the controls. This is in line with a study done in a CIA model in DBA/1 mice in which the abundance of *Alistipes* and *Streptococcus* was significantly reduced in arthritic mice when compared to nontreated mice. *Alistipes* has been associated with mucosal thickness; thus, it is likely to be decreased during dysbiosis (28).

*Helicobacter* has been associated with autoimmune diseases and inflammation (35, 36). The proportion of *Helicobacter* has been shown to be reduced in RA patients in China (26). Interestingly, the abundance of *Helicobacter* was reported to be reduced in multiple sclerosis (MS) patients and infection with *H. pylori* could be a potential protective factor for MS development (37). It has also been indicated that chronic infection with *H .pylori* induces Th2 and Treg proliferation which, in turn, increase anti-inflammatory cytokines and protect against autoimmune diseases (38). Hence, these are in line with our results which show a decrease in the abundance of the above-mentioned genera in comparison to the controls.

*Lactobacillus* was reduced in arthritic mice in comparison to the control mice. *Lactobacillus* can have either a protective or a harmful role in autoimmune diseases depending on the species. *Lactobacillus salivarius*, *Lactobacillus iners*, and *Lactobacillus reminis* have been identified in high numbers in RA patients and have been associated with the development of the disease (8). On the other hand, *Lactobacillus plantarum. Lactobacillus rhamnosus, Lactobacillus casei*, and *Lactobacillus helveticus* can act as probiotics and have been associated with anti-inflammatory immune responses (39).

The interaction between the intestinal microbiota and host immunity is dynamic and complex. During early life, colonization of the mucosal surfaces with commensal microbiota helps in the development and maturation of the immune system (40). In adulthood, a homeostatic environment in the intestine is maintained by regulating a basal level of inflammation and providing tolerance to self-antigens and commensal bacteria (41). Whenever dysbiosis occurs due to a combination of genetic and environmental factors, the composition and number of the different populations of the microbiota are affected and this leads to aberrant immune responses. This activates proinflammatory immune responses which, in turn, can affect further the microbiota and result in chronic inflammation, tissue injury, and diseases including autoimmune diseases (42). In this study, we identified the main immune responses that might be involved in the colons of arthritic mice by immunofluorescence. We examined two immune cell populations mainly IL-17A^+^ IFNγ^+^ cells and IL-17A^+^ FOXP3^+^ cells in the colons of these mice and in control mice. Cells that co-express IL-17A and IFNγ are more cytotoxic and potent and have been identified in a number of autoimmune diseases (43 – 45). There was no significant difference in the number of IL-17A^+^ IFNγ^+^ cells in the group that received collagen only and the group that received distilled water. However, the number of IL-17A^+^ IFNγ^+^ cells significantly increased in mice that received EBV DNA 6 days prior to collagen.

FOXP3^+^ CD4^+^ Treg cells are heterogeneous in gene expression, phenotype, and suppressive activities. A recent study identified FOXP3 +IL-17A^+^ Treg cells in the intestinal lamina propria of Crohn’s disease patients. Similar to Th17 cells, these cells could secrete IL-17, IL-22, IL-21, while expressing high levels of RORγt (46). In our study, both groups of arthritic mice had an increase in the number of IL-17A^+^FOXP3^+^ cells in comparison to the control group; however, the greatest increase was in the group that received EBV DNA 6 days prior to collagen. In addition, we showed that the highest increase in the number of cells that were triple positive for IL-17A, FOXP3, and IFNγ was in the group that received the EBV DNA in addition to collagen. A study by Afzali et al. (47) showed that the number of IL-17A^+^FOXP3^+^ cells in blood from RA patients was higher than in healthy controls (47). On the other hand, several studies reported an increase in IL-17A^+^FOXP3^+^ cells in the lymphocytes of IBD patients (46, 48). Furthermore, a number of studies have demonstrated that IL-17A^+^FOXP3^+^ cells produce IFNγ and IL-2 along with IL-17A (49 – 51). It has been proposed that ongoing autoimmunity in the EAE mouse model is likely to represent the defective function of Tregs and lose FOXP3 expression under inflammatory conditions and can be induced to express proinflammatory cytokines, such as IL-17 and IFNγ (IFNγ^+^ FOXP3^+^ IL-17A^+^) (52 – 54). In conclusion, these results might indicate that the increase in the number of triple-positive IFNγ FOXP3 IL-17A cells in the colons of arthritic mice could affect systemic inflammatory responses more so than non-IFNγ producing 17A^+^ FOXP3^+^ cells.

Following the observation that EBV DNA increases the incidence and severity of arthritis, upregulates immune cells involved in inflammation, and alters the diversity and composition of microbiota in an RA mouse model, we wanted to determine whether EBV DNA contributes directly to RA development. This was done by fecal transplantation from arthritic EBV DNA-treated mice to ones treated with antimicrobials followed by arthritis induction. We depleted the intestinal microbiota through antimicrobial treatment instead of using germ-free mice since we intended to use mice that had a mature immune system so as observe the effect of microbiota transplantation on arthritis development. Microbiota is essential for the development of the immune system postnatally, which, in turn, contributes to their regulation. Studies performed in germ-free animals showed that the microbiota plays a vital role in secondary lymphoid structure development (55 – 58). In addition, the lack of microbiota in germ-free mice is not exclusive to the intestinal microbiota; hence, by using antimicrobial-treated mice, we can examine the role of intestinal microbiota as a trigger for RA. In our study, we showed that mice that received feces from EBV DNA-injected arthritic mice had a higher incidence and severity of arthritis in comparison to mice that received feces either from collagen-treated mice or from distilled water-injected mice. Hence, this shows that the microbiota from EBV DNA-injected mice contributed to the higher incidence and severity of arthritis in the CIA mouse model. The incidence and severity of arthritis in the groups that received feces from collagen-treated mice and from distilled water-treated mice and then treated with type II chicken collagen were similar. This might be due to the likely possibility that both these groups would eventually have similar microbiota compositions since we are inducing arthritis using type II chicken collagen. These results suggest that the gut microbiota influences arthritis susceptibility.

EBV has been highly associated with the development of RA; attention has been previously given to various viral proteins as triggers of inflammation in this disease with little focus directed at the viral DNA which is shed upon viral reactivation. Microbial DNA has immunostimulatory properties and we have previously demonstrated significantly elevated levels of EBV DNA from subjects with RA compared to controls (14). Others have also reported similar findings detecting increased EBV DNA loads in RA patients (59 – 62). Increased levels of IL-17A have also been observed in subjects with RA (63, 64) and we have detected a positive correlation between EBV DNA loads and IL-17A levels in RA patients (14). Hence, we postulate that this viral DNA plays a role in the pathogenesis of the disease in human subjects similar to what we have observed in mice. This may occur at multiple levels including immunological changes that possibly result in a proinflammatory dysbiosis that then further contributes to the disease.

On the other hand, we previously assessed the effects of administering DNA from *Staphylococcus epidermidis* to the RA mouse model used in the study at hand. We observed that this bacterial DNA does not increase the incidence or severity of the arthritis features. In contrast, administering EBV DNA to the mice resulted in a significant increase in the incidence of arthritis in these mice as well as exacerbated the severity of the disease (15). Coupled with our previous observation that the *S. epidermidis* DNA does not increase the systemic levels of IL-17A in mice (13), observing no pro-arthritic effects for the bacterial DNA highly indicates that the properties of EBV DNA described herein are not universal to all types of microbial DNA. Moreover, we examined whether the viral DNA is transcribed in mouse cells resulting in the production of viral proteins that may mediate the observed immunostimulatory effects; however, we did not detect the production of viral RNA transcripts. This indicates that the proinflammatory effects are likely mediated by EBV DNA itself.

In conclusion, our study indicates that EBV DNA alters the composition and diversity of the intestinal microbiota in an RA mouse model resulting in dysbiosis, hence increasing the production of proinflammatory cytokines in the colons and joints, influencing the severity and incidence of the disease. A better understanding of the various factors involved in the development of RA will possibly help in creating individualized treatments, which might include targeting mediators triggered by viral DNA. Future studies should assess whether arthritic subjects with high EBV DNA loads have proinflammatory changes in their colonic microbiota. This may reveal possible therapeutic approaches that rely on targeting the mediators triggered by the viral DNA and may encompass microbiota modifications.

## Data Availability

The 16S rRNA sequencing raw data files are available via the National Center for Biotechnology Information (NCBI) BioProject with the accession number PRJNA972515.
